# Development and validation of a deep learning algorithm for discriminating glioma recurrence from radiation necrosis on MRI

**DOI:** 10.3389/fonc.2025.1573700

**Published:** 2025-06-06

**Authors:** Yu-Zhe Ying, Xiao-Hong Cai, Han Yang, Hua-Wei Huang, Dao Zheng, Hao-Yi Li, Ge-Hong Dong, Yong-Gang Wang, Zhong-Li Jiang, Zhu-Lin An, Guo-Bin Zhang

**Affiliations:** ^1^ Department of Neurosurgery, Beijing Tiantan Hospital, Capital Medical University, Beijing, China; ^2^ Institute of Computing Technology, Chinese Academy of Sciences, Xiamen, China; ^3^ School of Computer and Control Engineering, University of Chinese Academy of Sciences, Beijing, China; ^4^ Department of Critical Care Medicine, Beijing Tiantan Hospital, Capital Medical University, Beijing, China; ^5^ Departments of Pathology, Beijing Tiantan Hospital, Capital Medical University, Beijing, China

**Keywords:** glioma recurrence, radiation necrosis, convolutional neural network, magnetic resonance imaging, deep learning

## Abstract

**Purpose:**

Accurate differentiation between glioma recurrence and radiation necrosis is critical for the management of patients suspected of glioma recurrence following radiation therapy. This study aims to develop a deep learning-based methodology for automated discrimination between glioma recurrence and radiation necrosis using routine magnetic resonance imaging (MRI) scans.

**Method:**

We retrospectively investigated 234 patients who underwent radiotherapy after glioma resection and presented with suspected recurrent lesions during follow-up MRI examinations. Routine 3D-MRI scans, including T1-weighted, T2-weighted, and contrast-enhanced T1 (T1ce) sequences, were acquired for each patient. Among the analyzed cases, 192 (82.1%) were pathologically confirmed as glioma recurrence, while 42 (17.9%) were diagnosed as radiation necrosis. Various Convolutional Neural Network (CNN) models were employed to learn radiological features indicative of glioma recurrence and radiation necrosis from the MRI scans. Performance evaluation metrics, such as sensitivity, specificity, accuracy, and area under the curve (AUC), were used to assess the models’ performance.

**Result:**

Among the evaluated CNN models, ResNet10 demonstrated the highest sensitivity (0.78), specificity (0.94), accuracy (0.91), and an AUC value of 0.83. Additionally, the MresNet model achieved the highest specificity (0.980) but exhibited a relatively lower sensitivity (0.56). Another evaluated CNN model, Vgg16, showed a sensitivity of 0.56, specificity of 0.94, accuracy of 0.88, and an AUC value of 0.70.

**Conclusion:**

The proposed ResNet10 CNN model demonstrates promising performance on routine MRI scans, rendering it highly applicable in clinical settings. These findings contribute to enhancing the diagnostic accuracy for distinguishing between glioma recurrence and radiation necrosis using routine MRI.

## Introduction

Glioma, the most prevalent primary malignant brain tumor, is associated with a poor prognosis, particularly for high-grade gliomas (1, 2). Even after undergoing standard treatment, which includes surgical resection followed by radiotherapy and temozolomide chemotherapy, patients with glioblastoma multiforme (GBM) typically have a median survival of only 14.6 months (3). Radiation therapy has been shown to extend survival by up to 12 months (4). However, a notable complication following glioma treatment is brain radiation necrosis, which occurs in 3%-24% of patients within 2 years post-radiation therapy (5). Interestingly, radiation necrosis often coincides with the peak period of glioma recurrence (6). The clinical manifestations of radiation necrosis, such as the reappearance of initial symptoms, worsening neurological dysfunction, and progressive enhancement lesions with brain edema on radiographic images, closely mimic those of recurrent glioma (7). As a result, distinguishing between radiation necrosis and glioma recurrence based solely on routine magnetic resonance imaging (MRI) scans presents significant challenges (8). Accurate differentiation between these two conditions is critical for determining appropriate treatment strategies, as misdiagnosis can lead to severe consequences. Therefore, there is an urgent need to develop a reliable and user-friendly method for identifying radiation necrosis and tumor recurrence in glioma patients.

Recent studies have highlighted the utility of various advanced imaging techniques, such as perfusion-weighted imaging (PWI) (9), magnetic resonance spectroscopy (MRS) (10), diffusion-weighted imaging (DWI) (11), and positron emission tomography (PET) (12, 13), in differentiating radiation necrosis from glioma recurrence. These studies have identified several handcrafted radiomic features based on image intensity, shape, and volume characteristics associated with both conditions (14). However, the manual selection of these features may introduce bias, and manual segmentation of regions of interest (ROIs) is labor-intensive and time-consuming.

In previous studies, we observed promising results by integrating deep features into the radiomics model. However, most of these studies primarily focused on leveraging image information from single-modality MRI (15, 16). Additionally, deep neural network (DNN) models have been employed to enhance the classification of glioma recurrence versus necrosis, but they are limited by reliance on 2D routine MRI sequences and training on small, imbalanced datasets, which may lead to bias, overfitting, or undertraining. Gao et al. (17) introduced a novel DNN model for differentiating glioma recurrence from necrosis, yet it was constrained by a small dataset size and an imperfect patient cohort selection process. Santiago Cepeda et al. (18) developed a deep learning-based model (RH-GlioSeg-nnU-Net) for evaluating postoperative segmentation and resection of glioblastoma. Although this model demonstrated good performance across multiple datasets, it depends on manual or semi-automatic annotation, which may introduce certain biases. Moreover, some prior studies have reported on the application of deep learning models in glioma segmentation, finding that despite their strong performance, these models still face challenges in achieving precise segmentation in complex post-treatment backgrounds, particularly when handling post-treatment changes and the natural blurring of tumor boundaries (arXiv:2405.18368) (19–21).

In recent years, Convolutional Neural Networks (CNNs) have gained significant attention in the field of medical image classification and have achieved remarkable results (22, 23). CNNs are designed to mimic the mechanism of visual perception in organisms, resulting in state-of-the-art performance in visual analysis tasks and superior modeling capabilities.

Therefore, in this study, we propose a novel radiomics-based model for distinguishing between radiation necrosis and glioma recurrence. Our research leverages multimodal 3D routine MRI images and employs a 3D CNN architecture for experimentation. Notably, our study includes the largest cohort of cases compared to previous studies involving 3D imaging. Consequently, the proposed method demonstrates promising potential as a reliable clinical tool for accurately differentiating between glioma necrosis and recurrence.

## Methods

### Patient data and imaging protocol

**Figure 1:**
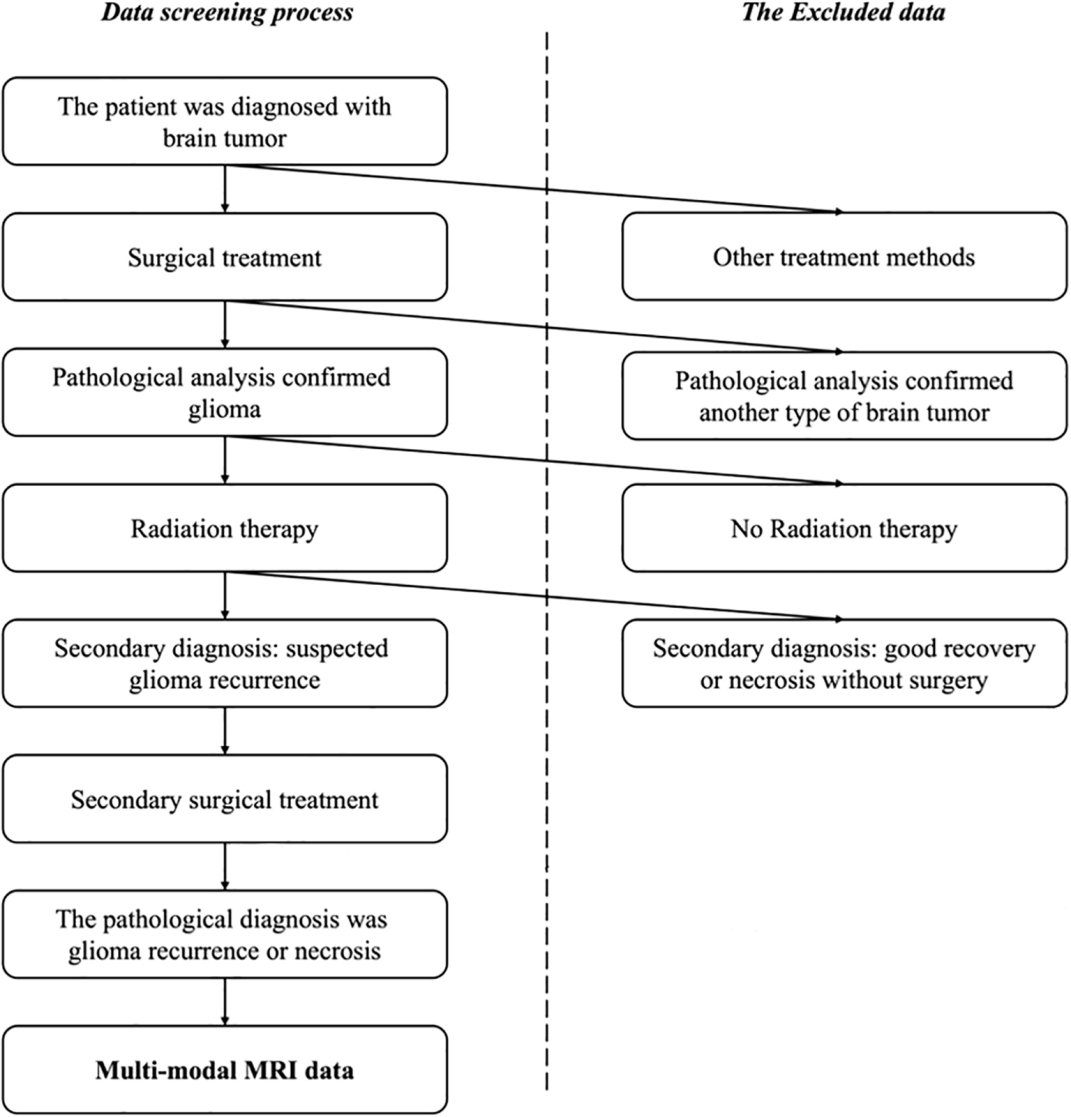
The selection process for the patient cohorts in this study. Patients with a confirmed pathological diagnosis, a history of radiotherapy, previous glioma diagnosis, and conventional MRI sequence data were enrolled while the patients without pathological examination results or missing conventional MRI sequence data were excluded, as well as patients with primary glioma or no history of radiation therapy.

This study included consecutive patients with glioma recurrence or radiation necrosis admitted to Beijing Tiantan Hospital, Capital Medical University, from January 2012 to December 2022. All procedures involving human participants were conducted in accordance with the ethical standards of the institutional and national research committees, as well as the 1964 Helsinki Declaration and its subsequent amendments or comparable ethical standards. The Institutional Review Board (IRB) of Beijing Tiantan Hospital, Capital Medical University, approved this study. Given the retrospective nature of the study, the IRB waived the requirement for informed consent. The inclusion criteria for participants are illustrated in **Figure 1**. All patients enrolled in this study had a confirmed pathological diagnosis of either glioma recurrence or radiation necrosis, a history of radiotherapy, a prior glioma diagnosis, and available conventional MRI sequence data. Patients without pathological examination results, missing conventional MRI sequence data, primary glioma diagnosis, or no history of radiation therapy were excluded.

Ultimately, a total of 234 cases were screened and included in the analysis. Among these, 192 cases were diagnosed with glioma recurrence, while 42 cases were diagnosed with radiation necrosis. The distribution of the collected data is summarized in **Table 1**.

**Table 1:** Demographic and clinical data of the patient cohorts enrolled in this study.

Characteristic	Training set	Test set	Total	P value
Simple size	176	58	234	
Age in years	46.55	46.98	46.87	0.797
Gender				0.290
Male	102	29	131	
Female	74	29	103	
Diagnosis of primary lesion				0.759
Glioma (grade 2)	65	20	85	
Glioma (grade 3)	37	12	49	
Glioma (grade 4)	67	23	90	
Unknow	7	3	10	
Diagnosis of recurrent lesion				0.199
Glioma (grade 2)	4	4	8	
Glioma (grade 3)	42	9	51	
Glioma (grade 4)	97	36	133	
Necrosis	33	9	42	

Continuous data were analyzed using t-tests, and categorical data between groups were compared using chi-square tests. A p-value < 0.05 was considered to indicate statistical significance.

MRI Acquisition: All subjects underwent MRI before surgery at the Center for Neuroimaging using a 3 Tesla MR scanner (imaging systems are detailed in **Table 2**) with a standard 8-channel head coil. The MRI acquisition protocol included the following sequences: anatomical 2D T1-weighted, T2-weighted, FLAIR, and contrast-enhanced T1-weighted imaging (T1ce). T1-weighted structural images were acquired with the following parameters: repetition time (TR) = 1900 ms; echo time (TE) = 8.6 ms; flip angle (FA) = 15°. T2-weighted structural images were acquired with the following parameters: repetition time (TR) = 4600 ms; echo time (TE) = 111.0 ms; flip angle (FA) = 12°. FLAIR images were acquired with the following parameters: repetition time (TR) = 8000 ms; echo time (TE) = 90 ms; inversion time (TI) = 2500 ms; flip angle (FA) = 10°. Contrast-enhanced T1-weighted images (T1ce) were acquired 5 minutes after intravenous injection of a paramagnetic gadolinium-based contrast agent (Gadolinium Diethylenetriamine Pentaacetic Acid, Gd-DTPA) at a dose of 0.2 ml/kg. The acquisition parameters for T1ce were identical to those used for the T1-weighted sequence.

**Table 2:** The imaging data acquired from the different magnetic resonance imaging systems.

Image system	Slice thickness, (mm)	Slice spacing, (mm)	Matrix size	Field of view, (mm)
Philips Medical Systems	5.0	6.0	272×179	100
SIEMENS	5.0	6.5	320×256	100
GE MEDICAL SYSTEMS	5.5	6.5	288×192	80
Philips	5.0	6.0	240×240	100

Each case’s data required a surgical diagnosis, wherein tumor or necrotic tissue was obtained during the operation, and an accurate label was assigned following histopathological analysis. One of the challenges addressed in this study was to develop a deep learning model capable of achieving high classification performance for practical medical diagnosis despite the presence of imbalanced datasets. In this study, the dataset was split into a training set and a test set in a ratio of 3.03:1.

### Histopathological diagnosis

The diagnosis of glioma recurrence and radiation necrosis was pathologically confirmed by the Department of Neuropathology at the Beijing Neurosurgical Institute. Fresh paraffin-embedded suspicious lesions were sectioned into 5-μm slices and stained with hematoxylin and eosin (H&E). If the original H&E-stained slides were of poor quality, new tissue blocks were prepared and restained. All available slices were blindly re-evaluated and reclassified by two experienced neuropathologists with over 10 years of experience in the field. For the diagnosis of radiation necrosis, only necrotic components were identified microscopically in the specimen, with no tumor tissue present. In contrast, the definition of tumor recurrence was the presence of glioma cells, regardless of whether necrotic components were observed. Although a mixture of tumor and necrosis is commonly encountered in clinical practice, our classification system can assist neurosurgeons in selecting the most appropriate treatment strategy. Patients diagnosed with tumor recurrence require surgical intervention, whereas those diagnosed with radiation necrosis generally do not require surgery.

### Data preprocessing

During on-site sampling, some scans may contain noise or have a varying number of slices. To standardize the data, we implemented the following data preprocessing pipeline: 1) Two-dimensional Dicom data corresponding to T1, T1ce, T2, and FLAIR image sequences were stacked along the z-axis to convert them into three-dimensional Nii format data. If a scan contained fewer than 32 slices for conversion, that case was excluded; 2) Nii data of different modalities were classified based on the modality information in the Dicom files; 3) Modal registration was prioritized in the order of T1 contrast enhancement (T1ce) > T1 > T2 > FLAIR; 4) A skull removal procedure was executed; 5) Multi-modal data were normalized in terms of size and pixel values. We applied the commonly used min-max normalization method for this purpose. In this method, the original pixel value is linearly transformed into the range [0, 1], and the formula is, $x^{\prime }=\frac{x-x_{min}}{x_{max}-x_{min}}$ where x is the normalized value, $x^{\prime }$ is the normalized value, $x_{max}$ and $x_{min}$ are the maximum and minimum values of the sample respectively.

It is worth noting that this study leveraged 3D MRI data and did not require additional lesion labeling by physicians, thereby significantly reducing their workload and enhancing the wide applicability of the method.

Overall, the T1 sequence primarily captures anatomical structures, the T2 sequence provides information on water content and lesion characteristics, the FLAIR sequence highlights the peritumoral region and reveals areas of edema, while the T1CE sequence further delineates intra-tumoral conditions and aids in differentiating between tumors and non-neoplastic lesions. Consequently, this study utilized multi-modal data as input to enable the network to learn richer visual features and achieve improved classification performance.

### Network and visualization

**Figure 2:**
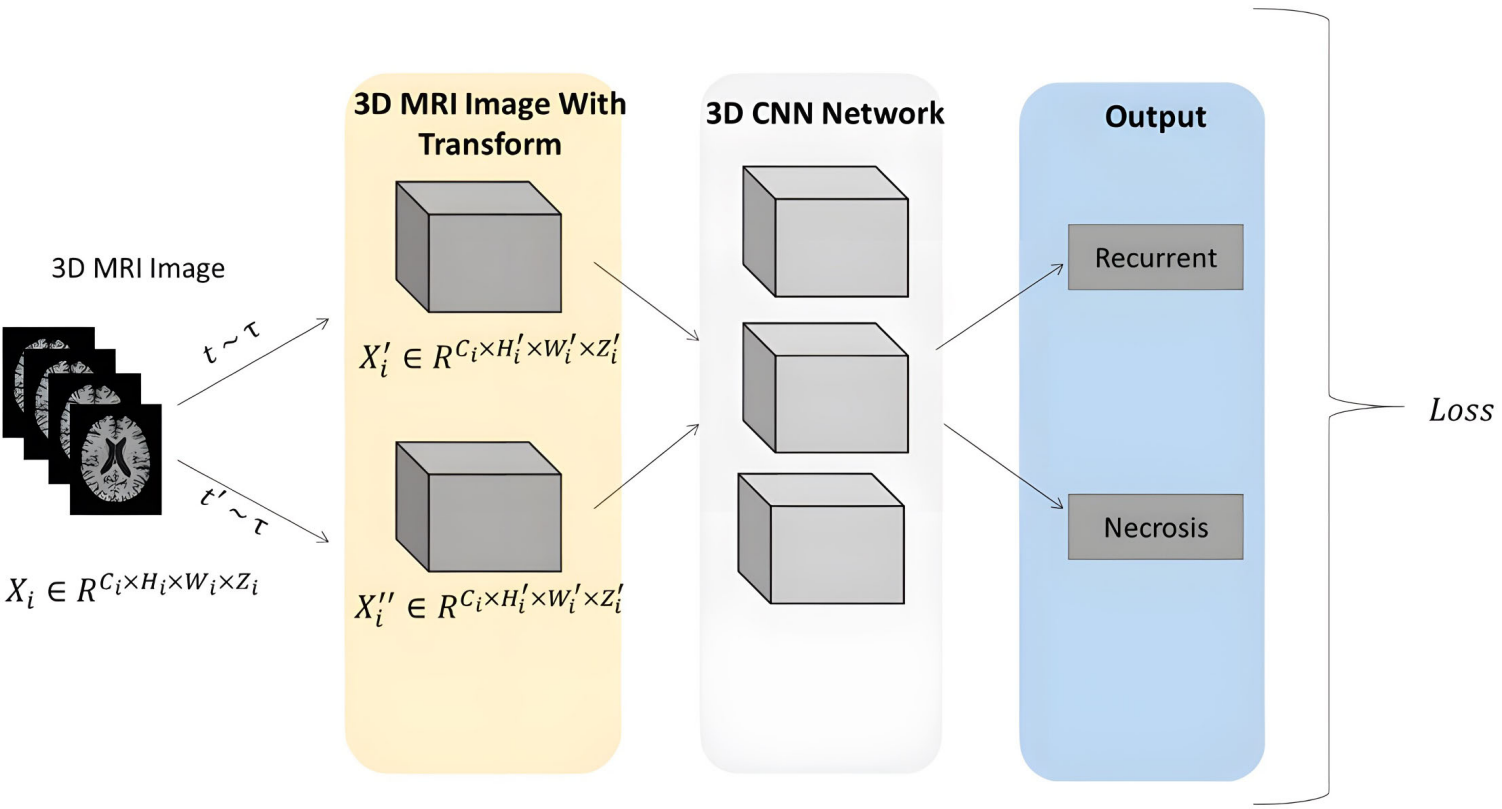
Overview of the proposed approach. The Pipeline of the CNN network was trained using 3D MRI sequences, abstracting low-level image features into high-level semantic features through cascaded layers of convolution and pooling. A fully connected layer is employed to perform the final classification task, yielding the diagnosis outcome. During supervised learning, the network’s classification results (radiation necrosis/tumor recurrence) are measured against the ground truth using loss calculation.

In recent years, Convolutional Neural Networks (CNNs) have been widely applied to medical image classification and have achieved remarkable performance. These CNNs take 2D or 3D medical images as input and progressively transform low-level image features into high-level semantic representations through a series of convolutional and pooling layers. Subsequently, a fully connected layer is utilized to perform the final classification task, thereby generating the diagnostic outcome. During supervised learning, the network’s predicted classification results (radiation necrosis vs. tumor recurrence) are compared with the ground truth via loss computation. The resulting loss is then backpropagated to guide the network’s parameter updates in the direction of minimizing the loss. Through multiple iterations, the model learns to identify critical features that distinguish tumor recurrence from radiation necrosis, features that often remain imperceptible to the human eye (see **Figure 2**).

To achieve two primary objectives—enhancing the network’s ability to learn visual features and reducing the workload of physicians—this paper proposes a multi-modal 3D CNN classification framework. We concatenate the four image sequences (T1, T2, T1ce, and FLAIR) along the channel dimension to construct a multi-modal 3D MRI sequence as input for our classification framework. The backbone of this framework can be adapted from any 2D convolutional neural network architecture. Specifically, we replace all 2D convolutional layers with 3D convolutional layers and reconfigure parameters such as kernel size, padding, and stride to accommodate 3D processing. After this modification, the classification network can accept 3D inputs and performing classification tasks. We conducted experimental evaluations using common convolutional neural network architectures, including ResNet10, ResNet50, VGG11, VGG16, and DenseNet121. Additionally, we compared the performance of different backbone structures under various input combination strategies. In all experiments, we set the learning rate to 1e-5, batch size to 2, and the number of epochs to 300.

Finally, the classification performance of the network is evaluated using several metrics, including accuracy, sensitivity, specificity, and the area under the receiver operating characteristic curve (AUC).

To further validate the classification performance of our model, we utilized Gradient-weighted Class Activation Mapping (Grad-CAM) to visualize the gradients of the last convolutional layer in the DenseNet121 backbone within our multi-modal 3D CNN classification framework.

Besides, the model’s discrimination relies on multi-modal MRI features, including: heterogeneous enhancement on T1ce, where irregular enhancement with perilesional edema is characteristic of recurrence, whereas necrosis often exhibits uniform ring enhancement; and peritumoral edema patterns on T2/FLAIR, where infiltrative edema is indicative of recurrence, while focal edema is more typical of necrosis. These findings are consistent with the experience and judgment criteria of radiologists.

### Evaluation metric

Four metrics—accuracy, specificity, sensitivity, and AUC—were utilized in this study to evaluate the model’s performance. Accuracy reflects the proportion of correctly predicted cases out of all cases, providing a comprehensive measure of the model’s overall performance. Due to the relatively small proportion of necrotic cases in the dataset and the critical importance of effectively differentiating necrotic cases, recurrence was defined as the negative class, while necrosis was defined as the positive class. Given the binary classification task of distinguishing glioma recurrence from necrosis, the model’s output consisted of the probabilities of each case being classified as recurrence or necrosis. These two probabilities summed to 1. The final prediction was determined based on the higher probability: if a case had a higher probability of recurrence, it was predicted as negative; conversely, if a case had a higher probability of necrosis, it was predicted as positive. Specificity refers to the proportion of correctly predicted negative cases (recurrence) out of all actual negative cases, while sensitivity refers to the proportion of correctly predicted positive cases (necrosis) out of all actual positive cases. Generally, predicting positive cases (necrosis) is more challenging due to their lower prevalence, and errors in predicting positive cases can have more severe consequences. Therefore, achieving higher sensitivity is desirable for the model. AUC, defined as the area under the Receiver Operating Characteristic (ROC) curve, serves as an evaluation metric for binary classification models. It represents the probability that the model ranks a randomly chosen positive case higher than a randomly chosen negative case. Higher AUC values indicate better model performance.

## Results

### Classification

Demographic characteristics are presented in **Table 1**, with a balanced distribution observed between the Training set and Test set (all P values > 0.05). **Table 3** provides a comprehensive performance analysis of different CNN models using single-modal 3D MRI sequences for classification. The T1 and T2 sequences demonstrate the highest accuracy, while the T1ce sequence exhibits the highest sensitivity. This can be attributed to the T1 sequence’s ability to capture detailed intracranial structural information and the T2 sequence’s strong correlation with water content, which aids in effective lesion characterization. Additionally, the T1ce sequence highlights valuable lesion features critical for distinguishing glioma recurrence from necrosis. Among the evaluated models, ResNet10 and ResNet50 achieved the highest accuracy of 0.91 (95% CI: 0.84–0.99) when using the T2 sequence as input. DenseNet121 and VGG16 achieved the highest accuracy of 0.88 (95% CI: 0.80–0.96) when employing the T1 or T1ce sequence as input. Notably, the diagnostic accuracy for negative cases (Recurrence) exceeded 90% across all three modal sequences, whereas the accuracy for positive cases (Necrosis) remained below 67%. This imbalance is likely due to the dataset distribution, underscoring the importance of enhancing model sensitivity for accurate identification of positive cases.

**Table 3:** Performance comparison of CNN-based models using single modal 3D MRI sequence as input.

Models	Scans	Acc(95%CI)	Sens (95%CI)	Speci (95%CI)	AUC (95%CI)
resnet10	t1	0.90 (0.82-0.97)	0.44 (0.15-0.77)	0.98 (0.88-0.99)	0.72 (0.60-0.83)
	t1ce	0.88(0.80-0.96)	0.56 (0.23-0.85)	0.94 (0.82-0.98)	0.70 (0.58-0.82)
	t2	0.91 (0.84-0.99)	0.44 (0.15-0.77)	1.0 (0.91-1.0)	0.70 (0.59-0.82)
densenet121	t1	0.88 (0.80-0.96)	0.67 (0.31-0.91)	0.92 (0.80-0.97)	0.79(0.68-0.89)
	t1ce	0.88 (0.80-0.96)	0.67 (0.31-0.91)	0.92 (0.80-0.98)	0.76 (0.65-0.87)
	t2	0.86 (0.77-0.95)	0.44 (0.15-0.77)	0.94 (0.82-0.98)	0.74 (0.63-0.85)
resnet50	t1	0.88 (0.80-0.96)	0.33 (0.090-0.69)	0.98 (0.88-0.99)	0.75 (0.69-0.86)
	t1ce	0.90 (0.82-0.97)	0.56 (0.23-0.85)	0.96 (0.85-0.99)	0.69 (0.58-0.81)
	t2	0.91 (0.84-0.99)	0.44 (0.15-0.77)	1.0 (0.91-1.0)	0.78 (0.67-0.88)
vgg11	t1	0.90 (0.82-0.97)	0.33 (0.090-0.69)	1.0 (0.91-1.0)	0.71 (0.60-0.82)
	t1ce	0.90 (0.82-0.97)	0.33 (0.090-0.69)	1.0 (0.91-1.0)	0.69 (0.58-0.81)
	t2	0.90 (0.82-0.97)	0.33 (0.090-0.69)	1.0 (0.91-1.0)	0.64 (0.51-0.76)
mresnet	t1	0.90 (0.82-0.97)	0.56 (0.23-0.85)	0.96 (0.85-0.99)	0.78 (0.67-0.89)
	t1ce	0.90 (0.82-0.97)	0.44 (0.15-0.77)	0.98 (0.88-0.99)	0.85 (0.76-0.94)
	t2	0.91 (0.84-0.99)	0.56 (0.23-0.85)	0.98 (0.88-0.99)	0.75 (0.63-0.86)
vgg16	t1	0.88 (0.80-0.96)	0.33 (0.090-0.69)	0.98 (0.88-0.99)	0.70 (0.58-0.82)
	t1ce	0.88 (0.80-0.96)	0.56 (0.23-0.85)	0.94 (0.82-0.98)	0.78 (0.68-0.89)
	t2	0.85 (0.75-0.94)	0.44 (0.15-0.77)	0.92 (0.80-0.97)	0.75 (0.64-0.86)

Acc, Accuracy; Sens, Sensitivity; Speci, Specificity; AUC, Area Under the Receiver Operating Characteristic Curve.

**Figure 3:**
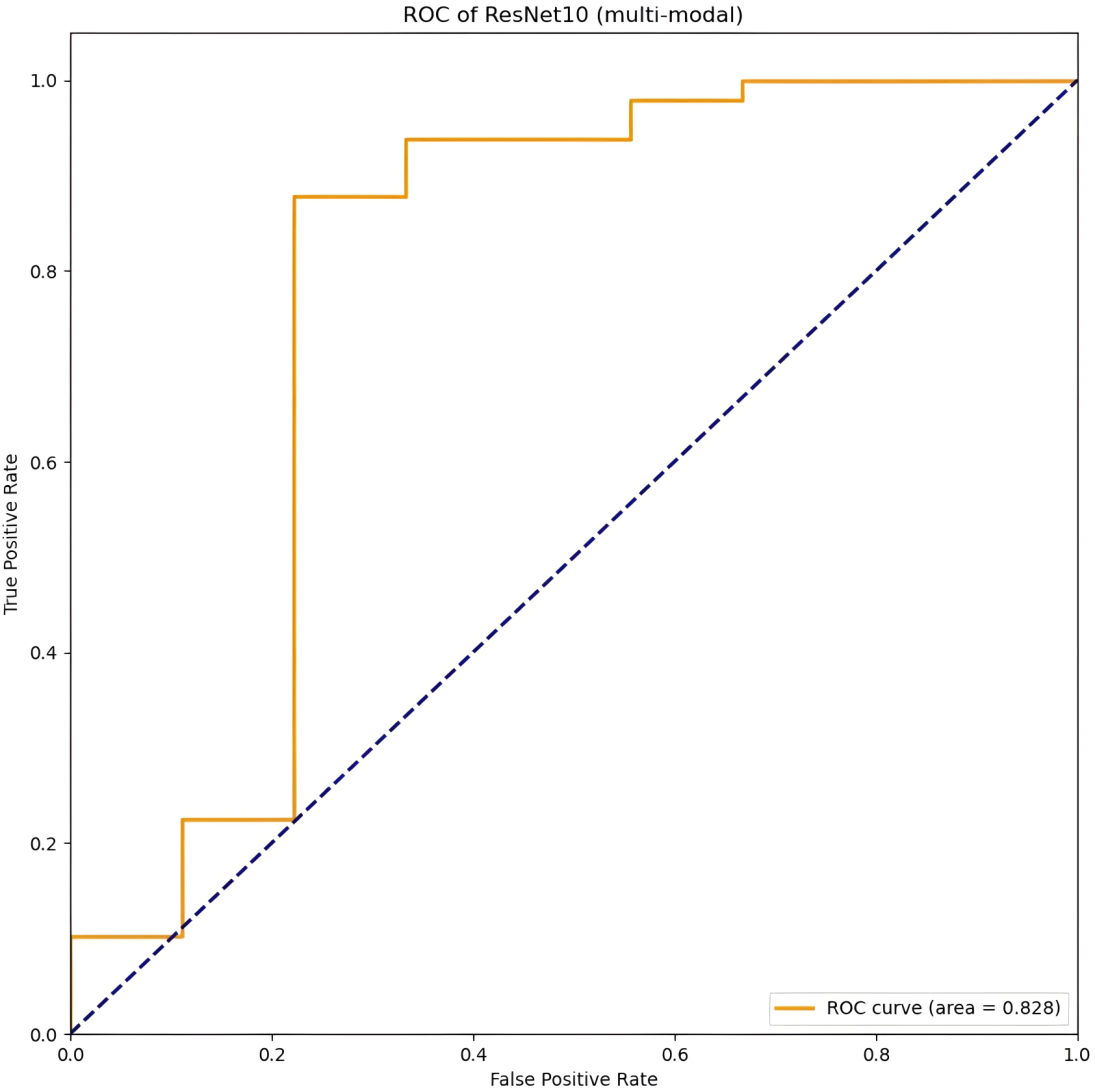
The best performance of the CNN model (Resnet10, t1, t2, t1ce) on multi-modal MRI in the image-based classification task.

**Table 4** presents the classification performance of a multi-modal 3D CNN model, which integrates fusion of 3D MRI sequences from all three modalities as input. Among the evaluated models, ResNet10 achieved the highest scores in terms of accuracy, sensitivity, specificity, and AUC, with respective values of 0.91 (95% CI: 0.84–0.99), 0.78 (95% CI: 0.40–0.96), 0.94 (95% CI: 0.82–0.98), and 0.83 (95% CI: 0.73–0.93) (ROC area shown in **Figure 3**). ResNet10 demonstrates an improvement in accuracy of 0.01 over DenseNet121 and ResNet50, and 0.03 over VGG11 and VGG16. Additionally, it achieves an AUC improvement of 0.03 over DenseNet121, 0.05 over ResNet50, 0.04 over VGG11, and 0.13 over VGG16. Notably, ResNet50, VGG16, and ResNet10 achieve the highest specificity score of 0.94 (95% CI: 0.82–0.98).

**Table 4:** Performance comparison of CNN-based models using multi-modal 3D MRI sequence as input.

Models	scans	Acc (95%CI)	Sens (95%CI)	Speci (95%CI)	AUC (95%CI)
**resnet10**	**t1, t1ce, t2**	**0.91** (0.84-0.99)	**0.78** (0.40-0.96)	**0.94** (0.82-0.98)	**0.83** (0.73-0.93)
densenet121		0.90 (0.82-0.98)	0.78 (0.40-0.96)	0.92 (0.80-0.97)	0.80 (0.70-0.90)
resnet50		0.90 (0.82-0.98)	0.67 (0.31-0.91)	0.94 (0.82-0.98)	0.78 (0.67-0.89)
vgg11		0.88 (0.80-0.96)	0.67 (0.31-0.91)	0.92 (0.80-0.97)	0.79 (0.68-0.89)
mresnet		0.91 (0.84-0.99)	0.56 (0.23-0.85)	0.98 (0.88-0.99)	0.73(0.62-0.84)
vgg16		0.88 (0.80-0.96)	0.56 (0.23-0.85)	0.94 (0.82-0.98)	0.70 (0.59-0.82)

Acc, Accuracy; Sens, Sensitivity; Speci, Specificity; AUC, Area Under the Receiver Operating Characteristic Curve.

The bolded values and models signify that this particular model demonstrates the optimal performance and the greatest efficacy among all the models presented.

We performed one-sample t-tests to evaluate the statistical significance of the predictive performance of each model when using the combined input of T1, T1ce, and T2 modalities, with the aim of determining whether the prediction probabilities were significantly higher than random guessing (0.5). As shown in **Table 5**, the statistical analysis results indicate that all models achieved p-values less than 0.05, accompanied by large absolute t-values. During testing, the proportion of the necrotic class was higher than that of the recurrent class. Therefore, we additionally reported independent p-values for each class, demonstrating that our model’s performance remained statistically superior to random guessing at the individual class level (**Table 6**). This finding provides robust statistical evidence that the proposed model in this study demonstrates significantly superior predictive performance compared to random chance in the classification task.

**Table 5:** The statistical analysis results of CNN-based models.

Models	p-value	t-value
resnet10	2.18*1e-7	8.33
densenet121	3.24*1e-6	5.15
resnet50	2.11*1e-6	6.37
vgg11	4.27*1e-5	5.88
mresnet	5.18*1e-7	7.04
vgg16	3.87*1e-5	4.72

**Table 6:** The independent p-values for each class.

Models	Necrosis	Glioma Recurrence
resnet10	6.97*1e-7	0.0024
densenet121	7.86*1e-6	0.0091
resnet50	4.31*1e-6	0.0087
vgg11	2.67*1e-5	0.0103
mresnet	8.18*1e-7	0.0042
vgg16	2.77*1e-5	0.0117

The fusion of multi-modal MRI sequences improved the feature learning and classification performance of the CNN model, as shown in **Tables 3**-**4**. By leveraging the anatomical structures captured by the T1 sequence, the lesion features highlighted by the T2 sequence, and the intra-tumoral characteristics revealed by the T1ce sequence, the model gains access to a richer and more multi-dimensional set of visual features. This integration ultimately enhances the model’s ability to classify and distinguish between different types of lesions more accurately and effectively.

### Visualization

**Figure 4:**
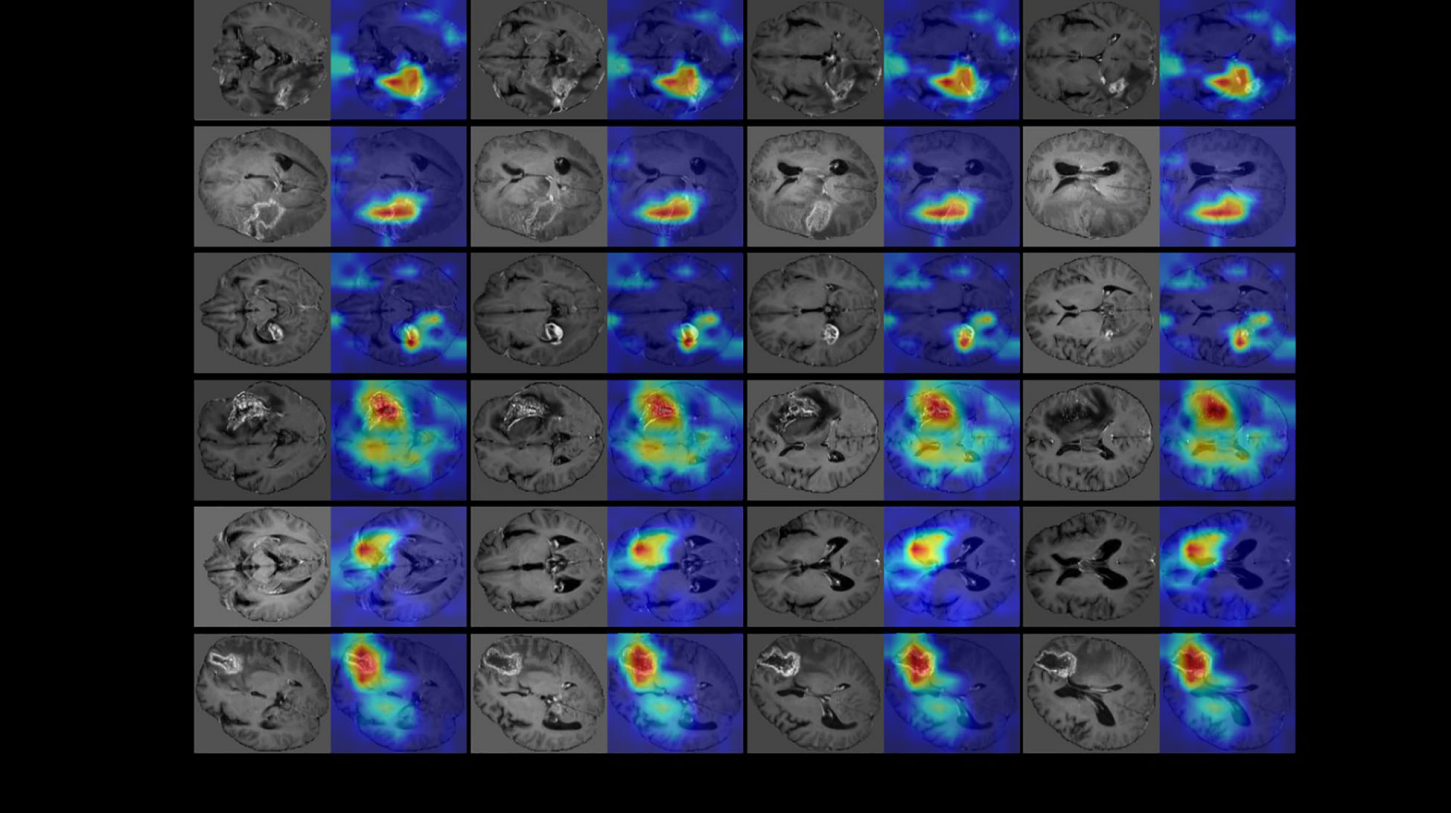
The visualization of the last convolutional layer in the DenseNet121 backbone of our multi-modal 3D CNN classification framework. To further demonstrate the classification performance of the model, we visualized the last convolutional layer’s gradient of the CNN model. We use GradCam algorithm (https://github.com/1Konny/gradcam_plus_plus-pytorch) visualization convolution level of output, its entropy diagram. Then we set the transparency of the entropy map and make it overlap with the original map, and the resulting effect is shown in the figure. The Grad-CAM highlighted the areas of highest network attention, with red indicating the highest attention and gradually transitioning to green, indicating reduced attention.

The visualization of the last convolutional layer in the DenseNet121 backbone is presented in **Figure 4**. Grad-CAM highlights the areas of highest network attention, with red indicating the highest attention and gradually transitioning to green, which indicates reduced attention.

Since our input was a 3D structure, we generated visualizations for each 2D slice. Notably, for recurrence cases, the network consistently focused on the central region of the tumor lesion, suggesting that it accurately captured and evaluated relevant features in that area. Similarly, in the case of necrotic lesions, the network’s attention was predominantly concentrated around and in close proximity to the center of the lesion. These observed attention areas in our visualizations provide evidence that the CNN model effectively diagnoses cases and achieves high diagnostic accuracy by leveraging relevant features.

This visualization using Grad-CAM demonstrates the ability of our model to focus on important regions within the input data, providing valuable insights into the decision-making process. Such visualizations help validate the model’s classification performance and enhance interpretability by highlighting the areas of highest network attention.

### Case illustration

**Figure 5:**
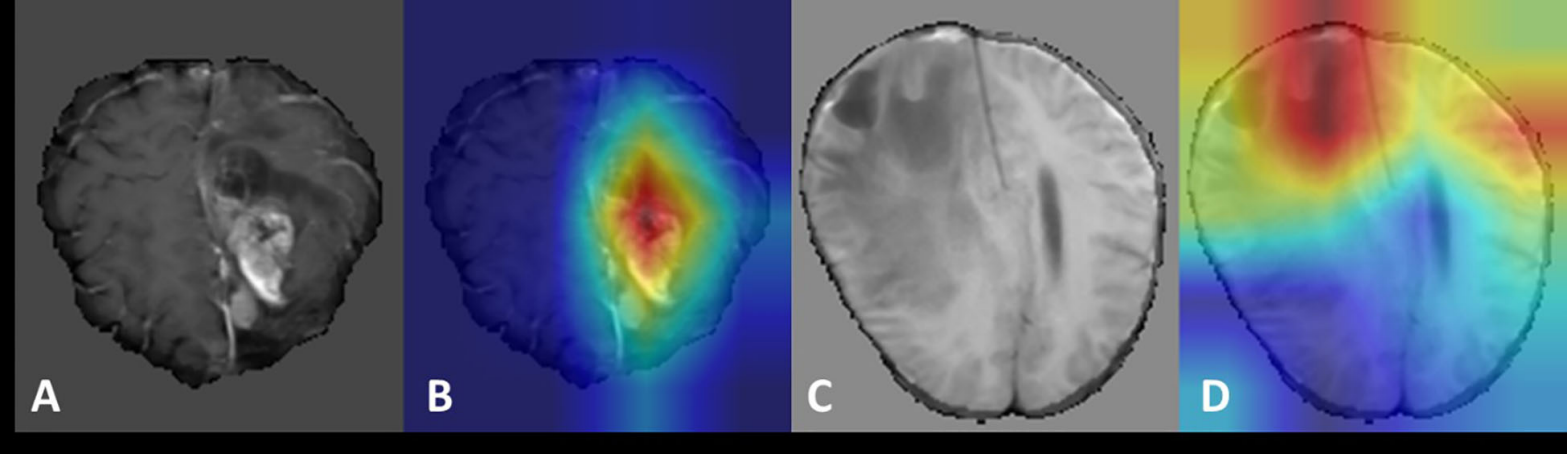
Visualization of recurrent glioma and radiation necrosis cases. **(A, C)** display the magnetic resonance images (MRI) after pre-processing such as skull removal used as model inputs for the recurrent glioma and radiation necrosis cases, respectively. **(B, D)** present the Grad-CAM visualizations for the recurrent glioma and radiation necrosis cases, respectively.

To illustrate the model’s workflow, we present two representative cases (**Figure 5**). After pre-processing steps such as skull stripping, multi-modal MRI images (T1, T2, T1ce, FLAIR) were input into the model, which outputs a numerical value. A value between 0–0.5 indicates radiation necrosis, while a value between 0.5–1 suggests recurrent glioma. Case 1 (Histopathology-confirmed recurrent glioma): A 52-year-old male with a history of GBM exhibited a heterogeneously enhancing lesion on T1ce. The model output a value of 0.92, and Grad-CAM highlighted the enhancing area (**Figures 5A, B**). Case 2 (Histopathology-confirmed radiation necrosis): A 45-year-old female presented with a ring-enhancing lesion. The model output a value of 0.11, with attention focused on the non-enhancing core (**Figures 5C, D**).

## Discussion

In our study, we employed multi-modal 3D MRI sequences from patients as input and conducted experiments using various commonly utilized convolutional neural networks, including ResNet10, DenseNet121, MresNet, VGG16, ResNet50, and VGG11. We compared the performance of different network architectures with varying input combination modes. Although prior studies have suggested that T1ce is the most informative MRI sequence for identifying necrosis (5, 6, 24), our findings demonstrated that when T1, T2, and T1ce modalities were fused as input, ResNet10 achieved the highest accuracy score of 0.914, which represents a remarkable achievement. Notably, when the three modalities were fused, ResNet10 also attained the highest sensitivity score of 0.778. These results indicate that CNNs can accurately identify radiation necrosis, even outperforming experienced neurosurgeons.

The proposed method demonstrates substantial clinical potential, as distinguishing glioma recurrence from radiation necrosis remains a critical challenge in clinical neuro-oncology (25). Misdiagnosing radiation necrosis as glioma recurrence may lead to unnecessary surgeries, while misdiagnosing glioma recurrence as radiation necrosis can delay effective treatment for glioma. Currently, the differential diagnosis of radiation necrosis and recurrent glioma relies on histopathologic analysis, which requires biopsy or open surgery for tissue collection. The method presented in this study enables accurate preoperative differentiation, assisting neurosurgeons in avoiding unnecessary invasive procedures and reducing risks for patients, as well as alleviating the economic burden. By analyzing the radiological features learned by the CNN models, our study provides valuable insights into the imaging characteristics of recurrent glioma and radiation necrosis. Consequently, these findings are likely to play a pivotal role in establishing guidelines for the differential diagnosis of recurrent lesions and in optimizing glioma follow-up strategies.

Compared with other deep learning methods, CNNs use 2D or 3D medical images as input, abstract low-level image features into high-level semantic features via convolutional and pooling layers, and accomplish the final classification task using fully connected layers to generate diagnostic results. Moreover, CNNs can compute loss based on ground truth, enabling backpropagation of loss and supervision of network parameter updates to minimize losses, thereby enhancing prediction accuracy. Importantly, the proposed method eliminates the need for time-consuming manual lesion delineation, which may introduce inter-reader variability. Furthermore, the performance of the proposed method surpasses that of previously reported methods (15, 16).

Recently, several studies have explored alternative models for distinguishing between necrosis and tumor recurrence. For instance, Gao et al. (17) proposed a novel deep neural network (DNN) model that uses 2D images as input and achieved higher performance, with the highest area under the curve (AUC) of 0.915. However, this model has certain limitations. It excludes patients who simultaneously suffer from both tumor recurrence and necrosis, which is an important consideration in clinical practice. Moreover, 2D images provide less information compared to 3D images. Additionally, another study reported a volume-weighted voxel-based multiparametric (MP) clustering method; however, the image-based segmentation of clusters was found to be less correlated with surgical specimens (26). Other existing techniques for differentiating recurrent glioma from radiation necrosis include perfusion-weighted imaging (PWI) (9), magnetic resonance spectroscopy (MRS) (10, 27), diffusion-weighted imaging (DWI) (11), and positron emission tomography (PET) (13, 28). Nevertheless, none of these techniques have demonstrated sufficiently high efficacy for routine clinical use. A meta-analysis of PWI and MRS revealed that the average relative cerebral blood volume (rCBV) in contrast-enhancing lesions was significantly higher in tumor recurrence than in radiation injury, and the average choline/creatinine (Cho/Cr) ratio was also significantly higher in tumor recurrence than in tumor necrosis, potentially improving the accuracy of differentiating between necrosis and recurrent tumor (29). Another study utilizing single-photon emission computed tomography (SPECT) and proton magnetic resonance spectroscopy (H1-MRS) demonstrated sensitivities of 88.9% for SPECT and 66.1% for MRS (30). However, these parameters only correlate with specific biological features, such as DWI with cell density and necrosis, CBV with vascular density, and MRS with metabolite concentration (26). Both aforementioned studies showed lower performance compared to the proposed methods. Furthermore, these existing methods are often expensive and not widely adopted in most Chinese clinical settings.

Previous studies on deep learning methods for this task share common limitations, such as the absence of pathological analysis and relatively small dataset sizes, which have impeded the clinical resolution of the differential diagnosis between tumor recurrence and necrosis (24). To the best of our knowledge, the dataset (N=234) utilized in this study constitutes the largest cohort among similar studies and incorporates pathologically confirmed diagnoses as ground truth labels, thereby enhancing its reliability for addressing this challenge.

However, the proposed method also has certain limitations. Our study is a retrospective analysis rather than a prospective one. Although our dataset is larger than most previous studies, it remains relatively small compared to generic image datasets commonly used in computer vision. Consequently, the confidence intervals for specificity and sensitivity are relatively wide. Furthermore, due to the retrospective nature of this study and the neurosurgeon’s experience in distinguishing necrosis from tumor recurrence, the CNN models were trained on an imbalanced dataset. Despite our efforts to split all cases into training and test sets (training:test = 3:1), the influence of the unbalanced data distribution could not be fully mitigated. The current experiments were conducted using a single-center dataset, and the model’s generalizability requires further validation on multi-center external cohorts. It would be beneficial to expand the sample size by incorporating data from other centers, particularly cases of radiation necrosis, to enhance and validate the proposed method.

Finally, imaging features specific to glioma subtypes and molecular genetic features, such as ATRX and 1p/19q status (31), as well as metabolomics indicators like phenylalanine, 2-glyceryl phosphate, lysine, and N-acetylaspartic acid (NAA) (32), were not included in this study. These aspects warrant investigation in future research. Due to time and computational resource constraints, direct comparisons with traditional radiomics approaches or hybrid AI methods were not performed in this study. Future work will involve benchmarking against baseline models to comprehensively evaluate the superiority of our approach.

While Grad-CAM visualizations preliminarily revealed the model’s focus on regions such as the tumor core and perinecrotic areas, systematic comparisons between these regions and radiologists’ diagnostic criteria (e.g., enhancing margins per RANO criteria) were not conducted due to time constraints and limited access to collaborative clinical expertise. Nevertheless, the observed attention patterns align with known imaging biomarkers of glioma recurrence and radiation necrosis. Our work provides a novel perspective on end-to-end deep learning for glioma imaging analysis. The preliminary results (high classification accuracy and Grad-CAM localization consistency) suggest the potential clinical utility of the proposed method.

## Conclusion

Our study demonstrated the effectiveness of multimodal 3D MRI-based CNN models in distinguishing recurrent gliomas from necrosis, outperforming other deep learning methods. The proposed method, which does not rely on lesion segmentation or handcrafted features, shows promising potential as a cost-effective and reliable tool for differentiating radiation necrosis from recurrent tumors. Given its high applicability in clinical settings, this deep learning approach holds significant value in improving diagnostic accuracy and enhancing patient outcomes. Further research and validation using larger and more diverse datasets, incorporating molecular and genetic features, will contribute to strengthening the robustness and generalizability of the proposed method.

## Data Availability

The datasets used and analyzed during the current study available from the corresponding author on reasonable request. Requests to access these datasets should be directed to Guo-bin Zhang, guobin_0912@sina.com.
